# Clinical and genetic analysis of pediatric catecholaminergic polymorphic ventricular tachycardia: focus on sinus bradycardia and neurodevelopmental disorders

**DOI:** 10.3389/fped.2026.1780382

**Published:** 2026-05-28

**Authors:** Lianfu Ji, Shiwei Yang, Qian Wang, Fan Yang, Jie Yin, Mei Chen, Jinlong Chen

**Affiliations:** Department of Cardiology, Children’s Hospital of Nanjing Medical University, Nanjing, China

**Keywords:** children, CPVT, genetic variants, inherited arrhythmias, neurodevelopmental disorders, personalized management, sinus bradycardia

## Abstract

**Background:**

Catecholaminergic polymorphic ventricular tachycardia (CPVT) is a rare hereditary arrhythmia with high mortality risk. Its clinical presentation is heterogeneous, and diagnosis is often delayed. While typically characterized by exercise-induced ventricular arrhythmias, CPVT can be associated with complex clinical features such as sinus bradycardia and neurodevelopmental disorders (NDDs), which complicate management. This study aims to improve awareness of CPVT among clinicians by summarizing the clinical and genetic characteristics of seven CPVT patients, with particular emphasis on these under-recognized comorbidities.

**Methods:**

Children with CPVT admitted to the Department of Cardiology of Nanjing Children's Hospital diagnosed with pathogenic variants from January 2020 to January 2024 were selected as the study subjects. We conducted a retrospective analysis of their clinical and genetic characteristics of these children.

**Results:**

A total of seven patients with CPVT were included, whose median age of onset was 7.7 (7.0, 8.8) years, and the longest diagnostic delay was nearly 8 years. All patients experienced exercise or emotional agitation prior to symptom onset: five presented with syncope, one with palpitations, and one with cardiac arrest. Notably, one patient exhibited sinus bradycardia on resting ECG, and two patients had comorbid neurodevelopmental disorders. The genetic tests revealed that four patients had variants in the *RYR2* gene. The p.R2420M and p.F4889L in *RYR2* had not been previously reported. The remaining three patients had variants in the *CASQ2*, *CALM2*, and *TECRL* genes, respectively. The patient with sinus bradycardia required an implantable cardioverter-defibrillator (ICD) for safe pharmacologic up-titration. After a mean follow-up period of (1.4 ± 0.7) years, five patients remained free of syncope, while one patient with an *RYR2* variant and neurodevelopmental comorbidity died suddenly.

**Conclusion:**

Here we analyzed the clinical and genetic characteristics of seven children with CPVT, and identified two novel variants in *RYR2*. It highlights the critical importance of recognizing and managing complex presentations, specifically sinus bradycardia and NDDs. Sinus bradycardia poses a therapeutic dilemma by limiting β-blocker use, often necessitating device therapy. Neurodevelopmental disorders signify a high-risk subgroup requiring aggressive management. Enhanced awareness and personalized risk stratification are essential for optimizing outcomes in pediatric CPVT.

## Introduction

Catecholaminergic polymorphic ventricular tachycardia (CPVT) is a hereditary, life-threatening arrhythmic syndrome. It is characterized by exercise- or agitation-induced bidirectional or polymorphic ventricular tachycardia, in the absence of structural cardiac abnormalities and with normal resting electrocardiograms (ECGs). The clinical manifestations of CPVT lack specificity; patients may present with palpitations, syncope, or even sudden cardiogenic death. The prevalence of CPVT is approximately 1/10,000, with a predominance in children, and the average age of first onset is 7–9 years (1). CPVT is considered highly malignant; untreated patients have been reported to have a mortality rate of 30% to 50% by 35 years of age (2).

The pathogenesis of CPVT is associated with impaired intracellular calcium handling. Variants in genes such as *RYR2* and *CASQ2* affect the release of calcium from the endoplasmic reticulum, leading to excessive calcium release during the diastolic phase of cardiomyocytes which disrupts the normal depolarization process and triggers ventricular tachycardia (3). This process can be aggravated by the release of endogenous epinephrine (4).

Since CPVT was first clinically described in 1975, our understanding of its genetic subtypes has advanced significantly. CPVT1, the most prevalent subtype accounting for 55%–65% of cases, arises from autosomal dominant mutations in the *RYR2* gene (5), whereas CPVT2, comprising 2%–5% of cases, is due to autosomal recessive *CASQ2* mutations (6). CPVT3, a rare infantile-onset subtype, was confirmed to be caused by recessive TECRL mutations (7, 8). Calmodulin-related CPVT caused by autosomal dominant mutations in *CALM1-3*, which frequently exhibits overlapping phenotypes with long QT syndrome (LQTS) and carries an exceptionally high risk of sudden cardiac death (9, 10). *TRDN*-related CPVT, caused by mutations in the triadin-encoding TRDN gene, may present with mild-to-moderate skeletal muscle weakness and exhibits a dual phenotype combining features of both CPVT and LQTS (11).

While the classic phenotype is well-described, CPVT can present with significant clinical heterogeneity such as sinus bradycardia and NDDs (12). Awareness of these complex presentations remains limited, contributing to diagnostic delays and suboptimal management. This study reports the clinical and genetic profile of seven pediatric CPVT patients, with a focused analysis on the diagnostic and therapeutic implications of concomitant sinus bradycardia and neurodevelopmental disorders.

## Materials and methods

### Patients

This study adhered to the guidelines of the Declaration of Helsinki (revised in 2013). Nanjing Medical University's ethics and plan review committee approved the study design for implementation. Children with CPVT admitted to the Department of Cardiology of Nanjing Children's Hospital with pathogenic variants from January 2020 to January 2024 were selected as the study subjects. Diagnosis was made according to the 2013 HRS/EHRA/APHRS Expert Consensus: Patients aged <40 years meeting one of the following criteria were diagnosed with CPVT: a. Unexplained exercise- or adrenaline-induced premature ventricular contractions (PVCs) or bidirectional/polymorphic ventricular tachycardia, with normal cardiac structure and ECG; b. Carrying of a CPVT-related gene variant or having family members with a CPVT gene variant.

### Whole-exome sequencing (WES)

Genomic DNA was isolated from peripheral blood samples of probands and their available parents using standard phenol-chloroform extraction protocols. Whole-exome capture was carried out using the IDT xGen Exome Research Panel v2.0, which covers approximately 23,000 protein-coding genes in the human genome. Enriched DNA libraries were amplified by PCR and subjected to 150-bp paired-end sequencing on the Illumina NovaSeq 6,000 platform, achieving an average sequencing depth of ≥100× and >98% coverage of target regions at 20 × depth. Variants with a minor allele frequency (MAF) > 1% were filtered out, and all identified variants were annotated using the Genome Aggregation Database (gnomAD). Candidate variants were further validated by Sanger sequencing, and their pathogenicity was evaluated according to the criteria of the American College of Medical Genetics and Genomics (ACMG).

### Outcomes and follow-up

Clinical data of the children were collected retrospectively, including past medical history, family history, results of ECG and treadmill tests, and treatments received. During follow-up, annual assessments via a combination of telephone calls and offline visits were conducted to ascertain whether the children experienced specific symptoms such as palpitations, syncope and cardiac arrest after treatment initiation.

### Statistical analysis

Descriptive statistical analyses were performed given the limited sample size. Continuous data are presented as mean ± standard deviation or median (interquartile range), as appropriate. Categorical data are presented as numbers and percentages.

## Results

### Baseline characteristics

A total of 7 pediatric patients (3 males, 4 females) were enrolled. The median age at symptom onset was 7.7 (7.0, 8.8) years. The median age at diagnosis was 8.8 (8.2, 14.1) years, resulting in a median diagnostic delay of 0.6 (0, 7.0) years, with the longest delay reaching 7.8 years (P6). All symptoms were exclusively triggered by physical exercise or intense emotional agitation, with no episodes occurring at rest or during sleep. None had a family history of sudden cardiac death, but two patients (P1 and P3) had first-degree relatives with unexplained syncope. Detailed baseline characteristics are summarized in Table 1.

**Table 1:** Clinical data of seven children with CPVT.

Number	Gender	Onset age (y,m)	Diagnostic age (y,m)	First symptom	Trigger	Family history	ECG	Treadmill test	Gene	Prognosis	Follow-up	NDDs	Therapeutic method
P1	F	7, 8	8,2	Syncope	Exercise	+	−	Exercise-induced PVCs, short VT	*RYR2*	Alive	26m	−	Propranolol
P2	M	8,2	8,2	Syncope	Exercise	−	−	Exercise-induced PVCs, short VT	*RYR2*	Alive	13m	−	Propranolol
P3	M	7,0	8,8	Syncope	Exercise/emotional stress	+	−	Exercise-induced PVCs, short VT	*RYR2*	Alive	33m	−	Propranolol
P4	M	9,2	9,7	Palpitation	Emotional stress	−	−	Exercise-induced PVCs, atrial flutter	*RYR2*	Sudden death	13m	Epilepsy, Mental retardation	Propranolol
P5	F	7,0	14,0	Syncope	Exercise	−	−	Exercise-induced short VT	*CASQ2*	Alive	14m	−	Propranolol + Propafenone
P6	F	6,4	14,2	syncope	Exercise	−	Sinus bradycardia	Exercise-induced PVCs, VT	*CALM2*	Alive	17m	−	Propranolol + ICD
P7	F	8,9	8,9	Cardiac arrest	Exercise	−	−	Not performed	*TECRL*	Vegetative state	5m	Mental retardation	Propranolol

F, female; M, male; y, year; m, month; NDDs, neurodevelopmental disorders.

### Clinical symptoms and auxiliary examinations

Five patients presented with exercise-induced syncope. P4, who had generalized tonic-clonic seizures since age 6 and abnormal EEG showing generalized epileptiform discharges, initially presented with recurrent palpitations triggered by emotional stress. P7 presented with out-of-hospital cardiac arrest while running, with initial ECG showing ventricular fibrillation. Both had confirmed NDDs: P4 had epilepsy and intellectual disability, while P7 had intellectual disability.

**Figure 1:**
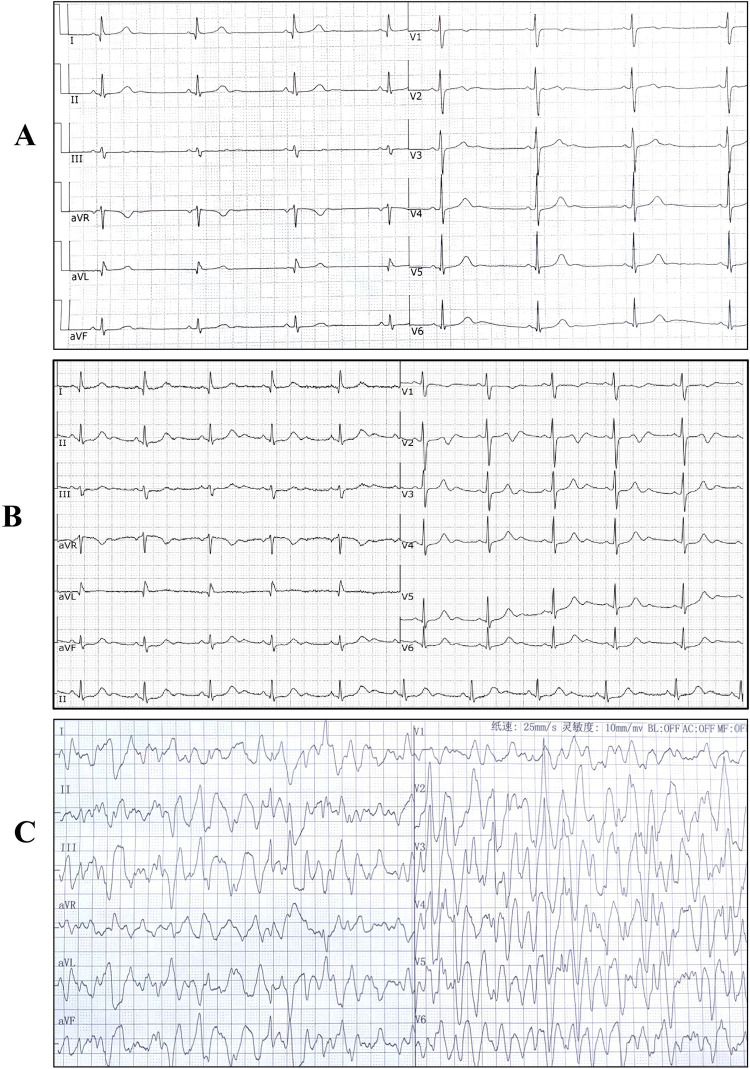
Representative resting ECG and arrhythmia recordings of P6. **(A)** Resting ECG showing sinus bradycardia (41 beats/min) with a QTc of 413 ms. **(B)** Resting ECG showing sinus rhythm (65 beats/min) with a QTc of 481 ms. **(C)** Treadmill exercise test showing PVCs and VT when the heart rate exceeded 100 beats/min.

All patients showed normal resting ECG findings without ST-T segment abnormalities, and QTc intervals were within normal limits in all individuals except P6. P6 harbored the *CALM2* p.N98S variant and showed mildly elevated resting QTc intervals of 413–488 ms, consistent with the long QT-like phenotype previously reported for this variant (10). In addition, P6 exhibited marked resting sinus bradycardia, with an average heart rate of 50 beats/min and a minimum of 33 beats/min (Figure 1). With the exception of P7, all 6 patients who underwent exercise treadmill testing had positive results, developing frequent PVCs or non-sustained VT at heart rates of 100–130 bpm. Notably, P4 also developed paroxysmal atrial flutter at peak exercise, which resolved spontaneously with rest. Transthoracic echocardiography was normal in all patients, with no structural abnormalities.

### Genetic testing

Genetic analysis identified pathogenic (P) or likely pathogenic (LP) variants in all 7 patients, in strict accordance with the 2015 ACMG standards. P1-P4 carried variants in the *RYR2* gene: p.E4005 V, p.R2474S, p.R2420M, and p.F4889L, which were located in the validated pathogenic hotspot regions III, II, II, and IV of the RYR2 protein, respectively. Specifically, the p.E4005V variant (c.12014 A > T) in P1, located in exon 90 (a well-recognized CPVT-related pathogenic hotspot of *RYR2*) (13), was classified as LP based on multiple lines of evidence: absence in the general population (gnomAD), consistent deleterious in silico predictions, and perfect match with the classic CPVT phenotype. Notably, the p.R2420M and p.F4889L variants have not been reported in previous literature or public genetic databases, thus our findings enrich the genotypic spectrum of CPVT.

The remaining 3 patients carried variants in other CPVT genes: P5 had compound heterozygous *CASQ2* variants, P6 had a *de novo CALM2* variant, and P7 had compound heterozygous *TECRL* variants. The biological information on specific CPVT-related gene variants is presented in Table 2.

**Table 2:** Genetic data of seven pediatric patients with CPVT.

Number	Gene	Variant	Variant origin	GnomAD	ACMG
P1	*RYR2*	c.12014 A > T p.E4005V	Mother	Absent	LP(PM1 + PM2_Supporting + PP2 + PP3_Moderate)
P2	*RYR2*	c.7422 G > C p.R2474S	*De novo*	Absent	P(PS3 + PM1 + PM2_Supporting + PM6 + PP2 + PP3)
P3	*RYR2*	c.7259 G > T p.R2420M	Father	Absent	LP(PM1 + PM2 + PM5 + PP2 + PP3)
P4	*RYR2*	c.14667 C > A p.F4889L	*De novo*	Absent	P(PS2 + PM1 + PM2 + PP2 + PP3)
P5	*CASQ2*	c.241 G > A p.A81T	Father	0.0000	LP(PM2_Supporting + PM3_Strong + PP3)
		c.232_c.234 + 2(IVS1)delGAGGT p.?	Mother	0.0000	LP(PVS1 + PM2_Supporting)
P6	*CALM2*	c.293 A > G p.N98S	*De novo*	Absent	P(PM6_Strong + PM1 + PM2 + PP3 + PS3_Supporting)
P7	*TECRL*	c.586 C > T p.R196*	Father	0.0000	LP(PVS1 + PM2_Supporting)
		c.587 G > A p.R196Q	Mother	0.0000	LP(PM2_Supporting + PM3_Strong + PP3)

P, Pathogenic; LP, Likely Pathogenic.

### Management and outcomes

All patients were advised to avoid strenuous physical activity and emotional excitement, and were prescribed long-term oral β-blockers. Propranolol was initiated at a dose of 1 mg/kg/day and titrated up to a maximum tolerated dose of 2–4 mg/kg/day, divided into 3 daily doses. P6, with preexisting sinus bradycardia, could not tolerate a target β-blocker dose (developed symptomatic bradycardia at 1.2 mg/kg/day). After shared decision-making with the patient's family, an ICD with backup pacing function was implanted 2 months after diagnosis. The pacemaker was programmed to a lower rate limit of 60 beats/min, allowing safe titration of propranolol to 2.5 mg/kg/day without symptomatic bradycardia. P5, with breakthrough arrhythmias on propranolol 3 mg/kg/day, required add-on propafenone at a dose of 15 mg/kg/day, divided into 3 daily doses.

After a mean follow-up period of 1.4 ± 0.7 years, 5 children had no recurrent syncope. P4, who carried a novel *RYR2* variant and had comorbid epilepsy/intellectual disability, died suddenly at home 13 months after diagnosis while playing with other children. She had been adherent to propranolol therapy (2.2 mg/kg/day) and had no prior breakthrough syncope. Post-mortem examination was declined by the family. P7 survived out-of-hospital cardiac arrest but suffered severe hypoxic-ischemic encephalopathy. He remains in a persistent vegetative state 5 months after the event, requiring mechanical ventilation and enteral nutrition support.

## Discussion

CPVT is a rare hereditary arrhythmia with high mortality risk, characterized by exercise- or agitation-induced bidirectional or polymorphic ventricular tachycardia. The initial symptoms of CPVT are diverse, including palpitations, syncope, cardiac arrest, seizure-like episodes, and chest pain. Among these, exercise- or agitation-induced syncope is the most common initial symptom of CPVT (14). In our study, five patients presented with syncope as their initial symptom. While exercise and agitation are the primary triggers for CPVT, it is worth noting that some patients may experience symptom onset during daily activities, at rest, or even during sleep (15).

The age at first onset of the seven patients was 7.7 (7.0, 8.8) years, which falls within the reported age of onset range for a Chinese pediatric CPVT cohort in the study by Yu Yan (14). The longest diagnostic delay was nearly 8 years for P6. Several factors may have contributed to this diagnostic delay. For example, parents with insufficient awareness might have mistaken syncope for hypoglycemia and thus failed to seek timely medical attention. Additionally, inexperienced clinicians with limited knowledge of CPVT may have difficulty diagnosing CPVT or even misdiagnose it as epilepsy. The exercise treadmill test is an important diagnostic tool for CPVT, but it should be noted that some CPVT patients may initially have negative results, which can turn positive as they grow older (2). Therefore, individuals highly suspected of having CPVT should undergo repeated treadmill exercise testing to avoid missed diagnoses or misdiagnoses.

CPVT is a genetically heterogeneous disorder, with multiple well-established pathogenic genes identified to date, including *RYR2, CALM1–3, CASQ2, TRDN,* and *TECRL*. Notably, phenotypic variations exist even among family members with identical gene mutations, including differences in age of onset, exercise-induced arrhythmia thresholds, and drug efficacy (16, 17). Four patients harbored *RYR2* variants, the most prevalent causative gene for CPVT1, with variants located in the four validated functional critical pathogenic hotspot regions of the RYR2 protein (17). Notably, we identified two novel *RYR2* missense variants (p.R2420M and p.F4889L) which expand the genotypic spectrum of CPVT and provide new data for genetic counseling and variant classification in clinical practice. In addition, we identified variants in *CASQ2, CALM2*, and *TECRL* in the remaining 3 patients, including a *de novo CALM2* p.N98S variant in the patient with sinus bradycardia (P6). Consistent with previous reports, this variant was associated with a mild LQTS-like phenotype with borderline QTc interval prolongation, highlighting the phenotypic overlap between calmodulinopathies and inherited arrhythmia syndromes (10, 17).

To prevent malignant arrhythmic events and sudden death in CPVT, a comprehensive treatment strategy is required-encompassing lifestyle management, pharmacotherapy, left cardiac sympathetic denervation (LCSD), and ICD. Currently, β-blockers remain the first-line treatment for CPVT, though studies have shown that approximately 25% of children with CPVT still experience syncope or cardiac arrest despite β-blocker therapy (18). Flecainide, a class I antiarrhythmic medication, has been shown to reduce ventricular arrhythmic events when used in combination with β-blockers among CPVT patients with persistent symptoms despite maximally tolerated β-blockers therapy. In cases where flecainide is inaccessible or not tolerated, propafenone can be considered as an alternative therapeutic agent (17, 19). In the case of P5, who initially received oral propranolol but still experienced frequent premature ventricular episodes during subsequent treadmill tests, propafenone was added to her therapeutic regimen; subsequently, she no longer experienced palpitations.

A key finding of our study is the critical clinical impact of concomitant sinus bradycardia on CPVT management, which remains an underrecognized therapeutic dilemma in clinical practice. Previous international studies have reported that approximately 20% of CPVT patients exhibit resting sinus bradycardia, which is an independent risk factor for adverse cardiac events (20, 21). The presence of this comorbid condition complicates pharmacotherapy, as it can limit the tolerance and optimal dosing of β-blockers. This scenario exposes the patient to risks from both the underlying CPVT and therapy-induced bradycardia. The treatment course of Patient P6 illustrates this common clinical dilemma in CPVT management. Our management approach resolved this conflict by implanting an ICD with backup pacing. The pacemaker ensures a minimum heart rate, preventing symptomatic bradycardia and enabling the administration of adequate, potentially high-dose β-blocker therapy. This strategy should be considered early for CPVT patients with significant resting or drug-induced bradycardia, as it optimizes both safety and therapeutic efficacy.

Another core highlight of our study is the identification of comorbid NDDs as a high-risk marker for adverse outcomes in pediatric CPVT. Previous large-scale international registry data first reported that the prevalence of NDDs is significantly higher in CPVT patients (8%) than in the general population (1%–3%) (12). In our cohort, two patients had confirmed NDDs, both of whom experienced devastating adverse clinical outcomes: one patient (P4) suffered sudden cardiac death despite adherent β-blocker therapy, and the other (P7) developed severe hypoxic-ischemic encephalopathy and persistent vegetative state after out-of-hospital cardiac arrest. These cases align with previous reports that NDDs, including intellectual disability and epilepsy, are associated with a significantly elevated risk of life-threatening arrhythmic events in CPVT patients (22). P4 harbored an *RYR2* C-terminal domain (CTD) variant, which confers a substantially higher risk of life-threatening arrhythmias in patients with CPVT (23). Furthermore, individuals with *RYR2* CTD variants are more likely to demonstrate resistance to β-blocker therapy (24). Collectively, these two cases suggest that CPVT patients comorbid with NDDs may be at an increased risk of adverse cardiovascular events and have a poorer long-term prognosis. Accordingly, the diagnosis of an NDD in a CPVT patient should prompt an immediate upgrade in risk stratification. Clinical management must adopt a more aggressive approach, including multidisciplinary team collaboration and serious consideration of primary prevention ICD implantation, even in the absence of breakthrough adverse events during pharmacological therapy.

We present a pediatric CPVT cohort highlighting the clinical and genetic diversity of the disease. Importantly, we identify sinus bradycardia and NDDs as critical features that complicate management and signify increased risk. Sinus bradycardia necessitates a tailored approach, often integrating device therapy to enable optimal pharmacological treatment. NDDs should alert clinicians to a potentially more malignant disease course, warranting intensified monitoring and therapy. Recognizing and systematically addressing these complex presentations is essential for improving personalized care and outcomes in children with CPVT. This is a retrospective, single-center study with a small sample size, limiting statistical power. The follow-up duration was relatively short for a lifelong disease. Larger, prospective multi-center studies are needed to validate the risk associated with these comorbidities.

## Conclusions

This study reinforces the genetic heterogeneity of CPVT, reporting two novel *RYR2* variants. Beyond expanding the genetic spectrum, our cohort underscores the clinical complexity introduced by sinus bradycardia and NDDs, which demand specific management considerations.

## Data Availability

The original contributions presented in the study are publicly available. This data can be found here: Genome Variation Map (GVM), Accession: GVM001422. Available at: https://ngdc.cncb.ac.cn/gvm/getProjectDetail?Project=GVM001422.
